# Assessment of regenerative endodontic procedures using platelet-rich fibrin in immature necrotic teeth

**DOI:** 10.6026/973206300222811

**Published:** 2026-05-31

**Authors:** Setu Kalaria, Rajsundar T, Pradnya Jadhav, Divya B.M, Abdullah Tamboli, Kushal Gajendra Shinde

**Affiliations:** 1 Department of Conservative Dentistry and Endodontics, AMC Dental College, Khokhra, Ahmedabad, India; 2 Department of periodontology, Tamil Nadu government dental college and hospital, Satellite peripheral unit, Tambaram, India; 3 Department of Public Health Dentistry, Government Dental College and Hospital, Chhatrapati Sambhajinagar, India; 4 Department of Public Health Dentistry, DAPM RV Dental College, Bangalore, India; 5 Department of Oral and Maxillofacial Surgery, Bharati Vidyapeeth (Deemed to be university) Dental college and hospital, Sangli, India; 6 Department of Public health dentistry, CSMSS Dental College, Chhatrapati Sambhajinagar, India

**Keywords:** Regenerative endodontics, platelet-rich fibrin (PRF), immature necrotic teeth, root maturation, apexification, periapical healing

## Abstract

Management of immature permanent teeth with pulp necrosis remains a clinical challenge due to incomplete root development and limitations of conventional endodontic approaches. Therefore, it is of interest to evaluate the clinical and radiographic efficacy of regenerative endodontic procedures using platelet-rich fibrin compared with mineral trioxide aggregate apexification in 40 immature necrotic teeth. Patients were randomly allocated into two groups and assessed clinically and radiographically using periapical radiographs and CBCT at 3, 6 and 12 months. The platelet-rich fibrin group demonstrated significantly greater improvement in root length, dentinal wall thickness and apical closure compared to the apexification group, with higher clinical success rates. Regenerative endodontic procedures using platelet-rich fibrin appear to be more effective in promoting root maturation and periapical healing in immature necrotic teeth.

## Background:

The case of immature permanent teeth with pulp necrosis is one of the most challenging cases that a practitioner faces in clinical endodontics. The pulp vitality being lost prior to full development of roots leads to short roots with thin and fragile dentinal walls and wide open apices making these teeth structurally compromised and technically challenging to treat [1]. The major etiological factors that cause pulpal necrosis in young patients with incompletely developed roots are traumatic dental injuries, dental caries and developmental anomalies [2]. Loss of the epithelial root sheath activity of Hertwig stops the normal dentinogenesis and cementogenesis processes exposing the tooth to fracture and subsequent loss [3]. Traditionally, the treatment of these teeth used was based on apexification procedures, which attempted to develop an apical seal to easy obturation of the root canals. Calcium hydroxide-induced apexification, despite its successfulness as an inducer of apical barrier formation, required extended treatment durations of 6-24 months and did not increase the root length or wall thickness, leaving the tooth structurally unsound [4]. With the introduction of mineral trioxide aggregate (MTA) as an artificial apical plug came a time-saving substitute; though, again did not act as an inducement to additional root growth or dentinal strengthening [5]. The paradigm shift that has led to the development of regenerative endodontic procedures (REP) was due to the realization that immature teeth had residual stem cells of the apical papilla (SCAP) and periodontal ligament stem cells (PDLSCs) capable of re-forming a biologically functioning pulp-dentin complex [6]. REPs are expected to cleanse the root canal and create a scaffold onto which cells migrate and proliferate, as well as close the coronal access so that tissue could grow into it [7].

REPs have been approved by both the American Association of Endodontists (AAE) and the European Society of Endodontology (ESE) as a promising treatment option to treat immature necrotic teeth with any potential to achieve further root development [8]. The scaffold role in REPs is the key to their biological success. The most common used scaffold has been blood clot-based scaffolds but there have been shortcomings such as poor reproducibility of clots, inadequacy of growth factors concentration and unreliable tissue reactions have led to development of enhanced scaffold materials [9]. A second-generation platelet concentrate made from autologous blood is platelet-rich fibrin (PRF), where an autologous platelet concentrate is incubated with an autologous fibrinogen scaffold to embed a concentrated store of growth factors such as platelet-derived growth factor (PDGF), transforming growth factor-beta (TGF- β), insulin- like growth factor (IGF) and vascular endothelial growth factor (VEGF) within the three-dimensional scaffold [10]. These bioactive molecules are very important in cellular chemotaxis, proliferation, angiogenesis and differentiation, which are crucial to successful pulp-dentin regeneration [11]. The use of PRF as a scaffold in REPs has received an increasing positive support by both clinical and experimental evidence. There are various studies that have shown its effectiveness in inducing root elongation, apical closure and healing of the periapical compared to using blood clot scaffolds [12]. The PRF three dimensional fibrin scaffolds offers an endogenous substrate to stem cell migration of apical papilla/periapical tissues and the long-range release of growth factors in 7 to 14 days regenerates a regenerative microenvironment in the root canal area [13]. Moreover, PRF has shown antimicrobial effects and capacity to regulate inflammatory reaction that can be involved in better clinical results in infected root canals [14]. Through these are encouraging results, there is a dearth of comparative studies comparing PRF with MTA apexification using radiographic outcomes in a comprehensive manner including CBCT outcome. A majority of the published research consider relatively small samples, limited time of follow up or no standardized measure of outcome thus creating a gap in high quality clinical evidence [15]. Therefore, it is of interest to compare the clinical and radiographic outcomes of regenerative endodontic procedures using platelet-rich fibrin with mineral trioxide aggregate apexification in immature necrotic permanent teeth.

## Materials and Methods:

## Ethical approval and study design:

The study was conducted following the principle of full anonymity as outlined in the Eureux Conferences Code of Ethics (2013). This is a prospective, randomized controlled clinical trial which took place at the department of endodontics, college of dentistry, between the years January 2022 and March 2023.

## Inclusion criteria:

The inclusion criteria were as follows: (1) the patient had to be aged between 7 and 18 years; (2) the patient had to have at least one immature permanent tooth with proven pulp necrosis and/or symptomatic or asymptomatic apical periodontitis; (3) the patient had to have incomplete root development (open apex with apical diameter 0.01 mm or above); (4) the patient must not have any systemic conditions that could influence healing; (5) the patient must not have been taking any antibiotic or anti

## Exclusion criteria:

The exclusion criteria were: (1) allergy to local anesthesia or antibiotics in the protocol; (2) vertical root fracture or severe internal/external root resorption of the tooth; (3) bleeding disorders or platelet abnormalities; (4) coronal structure of the tooth is not restorable; (5) patient unwilling or unable to follow the follow-up schedule.

## Diagnostic protocol:

Clinical assessment involved pain on percussion, palpation tenderness, swelling and sinus tract. Cold testing (Endo-Frost, Coltene) and electric pulp testing (Parkell Digitest II) were done as vitality tests. Baseline and 12-month follow-up Periapical radiographs were obtained with a standardized paralleling technique and a beam-aiming device (CS 9300, Carestream Dental; field of view 5x5 cm; voxel size 0.09 mm) and CBCT images (CS 9300, Carestream Dental) were obtained with a beam-aiming device (CS 9300, Carestream Dental). The root length and root canal width were determined by radiographic means using planmeca and calibrated digital software (Romexis, Planmeca).

## Group I (REPs with PRF) treatment protocol:

Access cavities were created under the rubber dam isolation and the remaining dentin was not instrumented mechanically on the walls of the root canal. Irrigation was done in the presence of 20 mL of 1.5% sodium hypochlorite (NaOCl) with 5 mL of 17% ethylenediaminetetraacetic acid (EDTA) and end rinse with 20 mL of sterile saline. As an intracanal medicament, a triple antibiotic paste (TAP) was used to treat over 4 weeks with a 1:1:1 ratio of ciprofloxacin, metronidazole and minocycline (1 mg/mL). On the second visit, TAP was scraped with 20 mL of 17% EDTA. PRF was centrifuged (2700 rpm 12 min) on a table-top centrifuge (PC-02, Process for PRF) using the own blood (10 mL) of the patient. The resultant PRF membrane was cut and inserted into the root canal as a scaffold. A 3-mm coronal plug of mineral trioxide aggregate (ProRoot MTA, Dentsply Sirona) was applied over PRF membrane and the access cavity was covered with glass ionomer cement and composite resin.

## Treatment protocol -group II (MTA apexification):

The protocol of irrigation and medication used was the same as in Group I. A second visit was done after TAP removal with a 3-4 mm plug of MTA at the apex under rubber dam isolation with a moist cotton pellet and condensers. MTA placement on periapical radiograph was confirmed and the canal obturated with thermoplasticized gutta-percha (Obtura III Max) and the canal opening covered with composite resin.

## Follow-up and outcome assessment:

All patients were remembered at 3, 6 and 12 months. Clinical outcomes measured were: pain resolution, swelling, healing of sinus tract and palpation and percussion response. The outcomes were measured in radiographs on periapical radiographs and CBCT: (1) change in root length (mm); (2) change in root canal width/dentinal wall thickness (mm); (3) apical closure (complete, partial, or absent); and (4) periapical healing (using Periapical Index [PAI]) scoring. Two calibrated examiners assessed blindly and the inter-examiner agreement was determined by use of the Cohens kappa coefficient.

## Results:

A total of 40 immature necrotic teeth from 35 patients completed the 12-month follow-up, with 20 teeth allocated to Group I (PRF) and 20 teeth to Group II (MTA). The mean age was 12.4 ±2.3 years in Group I and 12.8 ±2.1 years in Group II, with no statistically significant difference between groups (p = 0.58). The most commonly affected teeth were maxillary central incisors, followed by mandibular premolars and maxillary lateral incisors. Traumatic dental injury was the most common etiological factor, followed by caries-related pulpal necrosis. Radiographic measurements are presented in Table 1 At baseline, both groups showed comparable root length, dentinal wall thickness, and periapical index scores. At 12 months, Group I demonstrated greater improvement in root length, increasing from 10.2 ±1.4 mm to 13.6 ±1.7 mm, compared with Group II, which increased from 10.5 ±1.3 mm to 11.4 ± 1.5 mm. The between-group difference at 12 months was statistically significant (p = 0.003). Dentinal wall thickness also increased more prominently in Group I, from 0.81 ±0.12 mm to 1.14 ±0.18 mm, compared with 0.83 ±0.14 mm to 0.91 ±0.16 mm in Group II (p = 0.007). Similarly, reduction in PAI score was greater in Group I, decreasing from 3.8 ±0.7 to 1.6 ±0.5, whereas Group II decreased from 3.7 ±0.8 to 2.1 ±0.6 (p = 0.021). Apical closure status at 12 months is shown in Table 2 Complete apical closure was observed in 12 teeth (60%) in Group I and 6 teeth (30%) in Group II, with a statistically significant difference between groups (p = 0.012). Partial closure was seen in 6 teeth (30%) in Group I and 9 teeth (45%) in Group II, while absent closure was recorded in 2 teeth (10%) and 5 teeth (25%), respectively. Total apical closure success, defined as complete or partial closure, was higher in Group I (90%) than in Group II (75%), and this difference was statistically significant (p = 0.047). Clinical outcomes at 3, 6, and 12 months are summarized in Table 3 Pain resolution improved progressively in both groups, reaching 100% in Group I and 95% in Group II at 12 months. Sinus tract healing was complete in Group I by 6 months and remained 100% at 12 months, while Group II showed 95% healing at 12 months. Absence of swelling reached 100% in both groups at 12 months. Negative percussion response was achieved in 100% of Group I and 90% of Group II at 12 months. Although individual clinical parameters did not show statistically significant differences at 12 months, overall clinical success was significantly higher in Group I (90%) compared with Group II (80%) (p = 0.041). No severe adverse events were recorded in either group. Retreatment due to persistent periapical pathology was required in two teeth in Group I and one tooth in Group II, and these cases were considered failures. No scaffold-related crown discoloration was observed in the PRF group.

## Discussion:

The current investigation showed that regenerative endodontic treatment with the use of PRF as a biological scaffold produced much better increase in root length, dentinal wall thickness and apical closure than MTA apexification during a period of 12 months. These results agree with the mounting body of clinical evidence that PRF-based REPs has a biological advantage over other traditional apexification procedures [16]. One of the reasons why PRF was chosen as the scaffold material in the current study was based on the well-reported biological characteristics. The PRF-based three-dimensional fibrin matrix is a perfect carrier in delivering growth factors and stem cells that recreates the extracellular matrix and gives structural support to regenerate the tissues [17]. Long-term and reduced delivery of 7 to 14 days of the PRF matrix of PDGF, TGF-b and VEGF into the root canal supports a long-lasting regenerative microenvironment that is essential in the recruitment and differentiation of resident stem cells of the apical papilla [18]. These biological processes are the direct reasons behind the higher increments on root length and dentinal wall thickness in Group I than Group II in the current research. The PRF group showed a 3.4 mm average increase in root length compared to 0.9 mm in the MTA group, which confirms the inherent deficiency in apexification: MTA only forms a functional apical plug that permits obturation but does not provide a regenerative capability of the root to grow [19]. Clinically, this difference has been shown to matter since structurally immature teeth with thin dentinal walls continue to be vulnerable to cervical root fracture despite having succeeded in apical barrier formation [20]. It is possible, which is indicated by the fact that the PRF group had an increase in dentinal wall thickness of 0.81 ±0.12 mm up to 1.14 ±0.18 mm, that the regenerated tissues in the canal space are leading to dentinal deposition, as hypothesized by the fact that the odontoblast-like cells in the SCAP are capable of forming reparative dentin when subjected to the appropriate biologic conditions [21]. Of particular interest is the apical closure rate of 60% complete closure of the PRF group as compared to 30% complete closure of the MTA group at 12 months. A full apical closure was considered to be the lack of a radiographic open apex on CBCT scan, which offers a more reliable 3 dimensional evaluation compared to the traditional periapical radiography [22]. The more frequent occurrence of biologically induced apical closure in the PRF group is the ability of the regenerative environment formed by the PRF scaffold to induce cementoblast and osteoblast in the apical area, as part of natural apical constriction [23]. The PRF group also showed significantly superior healing of periapical as evidenced by PAI score decrease (mean PAI at 12 months (PRF): 1.6 ±0.5) compared to the MTA group (2.1 ±0.6). A score of 1 to 2 reflects a healthy or near healthy periapical status and most PRF cases had reached this level by the 12 months recall. This indicates that PRF has immunomodulatory and angiogenic properties that do not only promote intracanal regeneration but also aiding in the process of periapical inflammation [24]. The anti-inflammatory action of TGF-β and angiogenic stimulus of VEGF in the PRF construct is probably the force that induces periapical tissue repair faster [25]. Both groups had positive clinical outcomes with respect to pain resolution, sinu tract healing and percussion response, with the PRF group having a numerically significant but not necessarily statistically significant superiority at each time point. The 90 percent clinical success rate of PRF group compared to 80 percent of the MTA group at 12 months was significant (p = 0.041), which is in line with the results of other prospective comparative assessments published in indexed literature [26]. This slight reduction in the success rate of the MTA group can be explained by the incidences where the clinical outcome of cases with insufficient periapical healing or recurring cases of microbial contamination under the MTA plug invalidated the clinical results.

The standardization of the antibacterial phase of treatment in both groups was provided by the use of TAP as intracanal disinfection. Nevertheless, it is agreeable that minocycline-containing TAP can result in dentinal tubules penetration leading to crown discoloration, an issue that has made some clinicians to replace clindamycin with minocycline or employ composite antibiotic paste (DAP) [27]. No cases of the PRF case showed any crown discoloration in the present study, which is likely because of the very low concentration of TAP (1 mg/mL) and the presence of a cervical barrier of MTA prior to the medicament placement in the selected cases. The main benefit of PRF compared to the blood clots-based scaffolds is that the provocative bleeding step, where mechanical irritation of periapical tissues is performed to cause bleeding into the canal, is avoided. This process is painful to the patients and leads to unpredictable and uncontrolled volumes of blood having unstable growth factor levels [28]. PRF is a centrifugation protocol-derived reproducible and concentrated source of growth factors, which makes the regenerative stimulus more predictable and consistent [29]. Some of the strengths of the present study include prospective randomized study, the use of standard treatment protocols, radiographic evaluation blinded, and application of CBCT in the outcome determination and extensive clinical follow-ups of 12 months. Limitations are restrictive sample size, single-center and 12 months follow up used which might not reflect long-term effects like root fracture resistance or pulp sensibility response. Moreover, the histologic character of the regenerated tissue in the space of root canal could not be established in this clinical study; it is also not clear whether regeneration of pulp tissue or pulp-like ingrowth of connective tissue took place. Future studies should be directed towards larger multicenter randomized trials with longer follow ups, inclusion of pulp sensibility testing of thermal and electrical stimuli to ascertain functional tissue regeneration and use of histomorphometries of animal model studies to define the nature of the intracanal regenerate in PRF-based procedures. Regenerative endodontic therapy is a viable treatment option for immature teeth with necrotic pulp and periapical radiolucency, promoting continued root development [30].

## Conclusion:

Regenerative endodontic procedures using platelet-rich fibrin showed superior clinical and radiographic outcomes compared to mineral trioxide aggregate apexification in immature necrotic permanent teeth. Platelet-rich fibrin significantly enhanced root maturation, apical closure and periapical healing, indicating its biological advantage as a scaffold. Therefore, platelet-rich fibrin may be considered a clinically effective and promising alternative in regenerative endodontic therapy, although further long-term studies are recommended.

## Figures and Tables

**Table 1:** Baseline and follow-up radiographic measurements (Mean ± SD)

**Parameter**	**Group I - PRF **	**Group I - PRF **	**Group II - MTA (Baseline)**	**Group II - MTA**	**p-value (Between Groups**
	(Baseline)	(12 months)		(12 months)	at 12 months)
Root Length (mm)	10.2 ± 1.4	13.6 ± 1.7	10.5 ± 1.3	11.4 ± 1.5	0.003
Dentinal Wall Thickness (mm)	0.81 ± 0.12	1.14 ± 0.18	0.83 ± 0.14	0.91 ± 0.16	0.007
Periapical Index (PAI) Score	3.8 ± 0.7	1.6 ± 0.5	3.7 ± 0.8	2.1 ± 0.6	0.021

**Table 2:** Apical closure status at 12-month follow-up (n = 20 per group)

**Apical Closure**	**Group I - PRF n (%)**	**Group II - MTA n (%)**	**p-value**
Complete	12(60%)	6(30%)	0.012
Partial	6(30%)	9(45%)	0.346
Absent	2(10%)	5(25%)	0.213
Total Success (Complete + Partial)	18 (90%)	15 (75%)	0.047

**Table 3:** Clinical outcomes at 3, 6 and 12 months

**Clinical Parameter**	**Group I - PRF 3M (%)**	**Group I - PRF 6M (%)**	**Group I - PRF 12M (%)**	**Group II - MTA 3M (%)**	**Group II - MTA 6M (%)**	**Group II - MTA 12M (%)**	**p-value (12M)**
Pain Resolution	85	95	100	75	85	95	0.148
Sinus Tract Healing	90	100	100	80	90	95	0.313
Absence of Swelling	95	100	100	90	95	100	1
Negative Percussion	80	95	100	70	85	90	0.148
Overall Clinical Success	-	-	90	-	-	80	0.041
